# Revictimization and Mental Health Service Use in Intimate Partner Violence: A Comparison of Single and Multiple Reports Using Linked Police and Health Registers

**DOI:** 10.1177/00207640251379256

**Published:** 2025-10-09

**Authors:** Tomomi Hisasue, Marie Kruse, Johanna Hietamäki, Jani Raitanen, Visa Martikainen, Kimmo Suokas, Pekka Rissanen, Sami Pirkola

**Affiliations:** 1 Faculty of Social Sciences (Health Sciences), Tampere University, Finland; 2 Finnish Institute for Health and Welfare (THL), Helsinki, Finland; 3 Danish Centre for Health Economics, University of Southern Denmark, Odense, Denmark; 4 Department of Social Sciences, University of Eastern Finland, Kuopio, Finland; 5 UKK Institute for Health Promotion Research, Tampere, Finland; 6 Department of Psychology, University of Helsinki, Finland; 7 Tampere University Central Hospital, Finland

**Keywords:** intimate partner violence, mental health service use, revictimization, register study

## Abstract

**Background:**

Intimate partner violence (IPV) contributes to the development and severity of mental health problems, and pre-existing mental disorders are also associated with victimization. IPV is rarely a single event, and the consequences of revictimization appear to be more severe. However, little is known about patterns of mental health service utilization among individuals exposed to IPV revictimization.

**Aims:**

The study aims to estimate the associations between IPV revictimization and mental health service use over a 2-year period. Furthermore, we examine the association between pre-existing mental disorders and the risk of IPV revictimization.

**Methods:**

We conducted a register-based study including IPV victims identified from police reports in Finland, aged 19 to 54 years (*N* = 10,195), comparing single (*N* = 7,547) and multiple reports (*N* = 2,648) between 2016 and 2018. We applied the difference-in-differences method to estimate the effects of revictimization on mental health service utilization 1 year before and after the IPV event. Risk factors for revictimization were assessed using logistic regression, adjusting for sociodemographic factors.

**Results:**

Compared to the single event group, IPV victims with multiple reports exhibited higher mental health service utilization throughout the 2-year study period. In both groups, mental health service use peaked sharply around the time of the IPV event. The increase in mental health service utilization for IPV revictimization was approximately 8.0%, with a 0.9 percentage point rise following the initial IPV event. Pre-existing substance use disorders were significant predictors of revictimization for both men and women.

**Conclusion:**

Our main finding of higher mental health service use among IPV victims with multiple reports highlights the critical importance of early intervention. These results could reflect underlying poor socioeconomic conditions, pre-existing mental health conditions, and/or traumatic experience before the initial IPV report. Developing integrated services across mental health, social, and police services is crucial for providing preventative interventions to reduce further revictimization.

## Introduction

Intimate partner violence (IPV) is one of the most common forms of violence against women and is recognized as a serious public health issue worldwide (World Health Organization, 2021). Approximately 27% of ever-partnered women aged 15 to 49 years have experienced physical and/or sexual violence from an intimate partner in their lifetime (Sardinha et al., 2022). In Finland, the lifetime prevalence is even higher—34% among women and 18% among men (Attila et al., 2023). Victimization increases the risk of future victimization and leads to a range of mental health consequences, underscoring the importance of addressing this issue.

Systematic reviews have highlighted connections between mental health and IPV, including anxiety, depression, suicidality, or post-traumatic stress disorder (PTSD; Devries et al., 2013; Trevillion et al., 2012; White et al., 2024), as well as bidirectional associations: depressive symptoms predispose to subsequent IPV, while recent IPV experience increases the occurrence of risky substance-related behaviors (Bacchus et al., 2018; Devries et al., 2013). Furthermore, exposure to violence before “recent” IPV, such as adverse childhood experience, may contribute to both mental health consequences (Abate et al., 2024) and susceptibility to IPV (Zhu et al., 2024).

Although there is no uniform definition, revictimization commonly refers to a repeating experience of violence by the same or different partners (Bellot et al., 2024). The cumulative impact of traumatic events from repeated IPV seems to increase the need for mental healthcare. However, it is currently not known how lifetime exposure to IPV revictimization influences mental health, as existing research does not fully assess the duration, severity, or differences across study populations.

Despite growing research interest, studies on the outcomes of revictimization remain scarce (Bellot et al., 2024). Notably, between 23% (Vatnar & Bjørkly, 2008) and 56% (Alexander, 2009) of women who left their abusive partners were later exposed to violence in new relationships, but the risk factors for revictimization are not fully understood. Systematic reviews have highlighted that, among mental health factors, only substance use is considered a strong risk factor for IPV revictimization (Kuijpers et al., 2011; Ørke et al., 2018). Among other factors, child maltreatment is the most consistently identified risk factor, while younger age, employment status, recent physical violence, and the severity of previous violence have shown mixed results (Bellot et al., 2024; Ørke et al., 2018).

Understanding patterns of mental health services among individuals exposed to IPV revictimization is crucial. Previous studies have shown increased utilization of mental health-related emergency department services among IPV victims (Papalia et al., 2024; Rasmussen et al., 2021) and higher health-related costs (Hisasue et al., 2024; Kruse et al., 2011; Rivara et al., 2007). However, IPV victims often face barriers to accessing formal healthcare services and are unwilling to disclose their victimization when the perpetrator is someone in a close relationship (Christ et al., 2022; Hullenaar & Frisco, 2020; Pietiläinen et al., 2022). Earlier research on IPV revictimization has been limited, primarily relying on cross-sectional designs and focusing exclusively on women. This study addresses these gaps by utilizing a unique register-based dataset that links police records with health registers, including both men and women. Although IPV can affect all genders, this study defines it as a binary variable due to limitations in the available register data.

The current study aims to examine how mental health service utilization changes before and after the initial IPV event for individuals who were exposed to IPV as an isolated event compared to those who were exposed to repeated IPV, based on cases reported to the police. We hypothesize that individuals with multiple reports, indicating revictimization, use more mental health services. Second, we examine the association between pre-existing mental health conditions and identify the risk factors for IPV revictimization.

## Materials and Methods

In this population-based register study, we employed a quasi-experimental difference-in-differences (DID) approach to examine mental health service utilization 1 year before and after the initial IPV event reported to the police between 2016 and 2018. The study included individuals aged 19 to 54 years at the time of the reported offense. The administrative data used in this study contained information on IPV-related offenses based on police reports, while sociodemographic information was linked to health registers through personal identification codes.

### Study Population

#### Intimate Partner Violence

The original data were derived from nationwide offense records in the police information system maintained by Statistics Finland. The police records did not include background information on the specific relationships between suspects and victims or other sociodemographic details. However, Statistics Finland linked information on victims and suspects to other registers (e.g. residential ties), using unique personal identification codes, and created a dataset to determine the relationship between suspects and victims (Statistics Finland, 2024). Based on the data, it was possible to identify the relationship between a suspect and a victim as (a) a current spouse or cohabiting partner, (b) a former spouse or former cohabiting partner, or (c) having a common child with the suspect. We defined IPV cases as those meeting any of these three relationship criteria. From our dataset, we could not determine whether the perpetrator was the same or different individuals. We included all offenses categorized as physical (e.g. assault and petty assault), sexual, and personal such as robberies, deprivations of personal liberty, menaces, and persecution.

#### Revictimization

Individuals were categorized into two groups based on their reporting patterns from 2016 to 2018: (1) single report, referring to individuals with an isolated report of victimization during the study period; (2) multiple reports, referring to individuals with several reports of victimization within the same time frame. For this study, we broadly defined revictimization as having multiple reports, regardless of whether the offenses were of the same or different types over time. Reports of different types of offenses may occur on the same date or at different times. When an individual had two or more reports, we selected the earliest incident within the study period as the initial IPV event. We then categorized individuals with revictimization as the treatment group and those with an isolated report as the control group.

**Figure 1.**
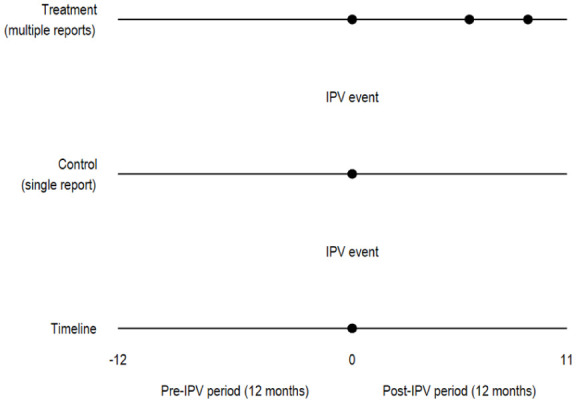
Illustration of the observation period and the initial IPV event to distinguish between single (isolated) and repeated IPV victim groups.

We used an event window of 365 days before and 365 days after the initial IPV event, covering a 2-year observation period. The dataset included the report date (the date the offense was reported to the police) and the offense date (the date the offense occurred). In some cases, a time lag existed between these two dates, and the report could also include multiple offense reports on the same dates (Figure 1).

We limited the study population to individuals whose earliest offense date as IPV victims occurred between 1 January 2016 and 31 December 2018 and who were aged 19 to 54 years on the offense date, regardless of report date. Individuals who reported exposure to IPV occurring under the age of 19 years were excluded to maintain our study focus on IPV, given that younger individuals may not have full autonomy in accessing healthcare services. The rationale for this time frame was to allow for analysis of healthcare utilization 1 year before and 1 year after the initial IPV event. We excluded individuals who reported IPV victimization occurring before 1 January 2016. This time frame ensured at least 1 year without police-reported victimization to establish a baseline.

### Outcomes

#### Mental Health Service Utilization

We linked the primary data at the individual level with two nationwide registers: (1) the Finnish Care Register for Healthcare and (2) the Register of Primary Healthcare Visits. Our primary outcome, mental health service use, is defined as any healthcare visit for mental disorders, including contact with secondary care psychiatric inpatient or outpatient services or primary care, with a diagnosis of mental disorder (ICD-10 diagnoses F00–F99) or International Classification of Primary Care-2-chapter “P” category during the study period. Noting that some health register entries overlapped, we applied a method to address these overlaps and accurately identify mental health service utilization (Suokas et al., 2024).

Any mental health service contacts are coded as binary variables and were aggregated to an individual monthly level. We used binary outcome variables instead of count variables, as substance abuse disorders often involve intensive healthcare visits. Using binary outcomes may reduce bias in assessing the effects of IPV revictimization.

When investigating the associations between IPV revictimization and pre-existing mental disorders, the following categories were examined: any mental disorders (F00–F99 and ICPC2 “P”), substance use disorders (SUD; F10–F19), depression (F32–F39), and anxiety (F40–F42, F44–F45, F48; Supplemental Table 1).

#### Covariates

We used the following individual-level sociodemographic variables to examine health service use and risk factors for IPV revictimization. Gender and cohabitation status (same or separate residence) between the victim and the suspect were included as binary variables. Age (19–29, 30–39, and 40–54 years) and education (primary, upper secondary, and tertiary) were categorized into three groups. Occupational status was classified as “employed” or “not employed,” with the latter category including unemployed, and all others being outside the labor force. Education and occupation were measured at the end of the year preceding the initial violence event.

For information on education and occupation, 65 individuals had missing values at the end of the year preceding the initial victimization. For these cases, values from the end of the event year were used when available; otherwise, information from the following year was applied. Finally, data for eleven individuals remained missing, and these individuals were excluded from the multivariable analyses.

#### Statistical Analysis

First, we applied a DID approach, a quasi-experimental method that compares changes in outcomes over time between two groups. While our analysis used individual-level data, our primary focus was on group-level differences in the effect of revictimization over time. Each individual’s observation period was aligned relative to their specific event date, making the time intervals comparable across individuals, regardless of when the event occurred between 2016 and 2018 (see Supplemental Equation 1). We estimated parameters using linear probability models (ordinary least squares: OLS), with outcome variables coded as binary. To account for within-individual correlation, we used clustered standard errors. OLS estimates were presented as pre-post changes in percentage points. We also calculated the relative change in percent by dividing the absolute estimate by the baseline value of the treatment group prior to the initial violence-related health contact and multiplying by 100 in crude models.

Next, we investigated the associations between pre-existing depression and SUD and the risk of revictimization, using logistic regression models stratified by gender. Since the outcome was revictimization (yes/no) defined as more than one IPV report within the post-IPV event period, only the pre-period before the initial IPV event was included the analyses. Predictor variables included age, education, occupation, and cohabitation status. We reported adjusted odds ratios (ORs) with 95% confidence intervals (CIs).

We also performed all analyses stratified by gender. The rationale for this sub-group analysis was based on previous research suggesting gender differences in violence revictimization among patients with depressive disorders (Christ et al., 2022) or previous psychiatric disorders and IPV victims (Ahmadabadi et al., 2020). All analyses were conducted using Stata, version 18 (StataCorp, 2023) and R, version 4.3.1 (R Core Team, 2023), respectively.

## Results

The final sample consisted of 10,195 individual IPV victims, including those with a single report (*N* = 7,547) and those with multiple reports (*N* = 2,648). Each IPV victim contributed 24 observations to the panel dataset, resulting in a total of 244,680 observations over the 24-month study period (12 months before and after the initial IPV event). Overall, 43.6% of them had at least one contact with mental healthcare during the study period.

Table 1 presents descriptive statistics for the single report group (74.1%) and the multiple reports group (26.0%) during the year of the IPV event. The majority of IPV victims were women in both groups, with an even higher proportion in the multiple reports group. Approximately 47% of the total population in both groups were non-employed and had secondary education as their highest level of attainment. The most common offense type in both groups was physical assault. Personal offenses, particularly menace, were more common among those with multiple reports than among those with a single report. IPV victims with multiple reports showed a higher prevalence of pre-existing mental disorders in the 12 months before the IPV event compared to those with a single report. Depression was the most common mental disorder in both groups (multiple reports 11.7% vs. single report 9.3%). Over the entire study period, inpatient care accounted for approximately 2.1% of all mental health service use, while psychiatric hospitalization accounted for 0.3%, indicating that IPV victims predominantly utilize outpatient visits.

**Table 1.** Descriptive Statistics of IPV Victims With Single and Multiple Reports During the Year of the Initial Report.

Variable	Multiple reports (treatment)		Single reports (control)	
	*N* = 2,648		*N* = 7,547	
	*N*	%	*N*	%
Gender				
Men	339	12.8	1,937	25.7
Women	2,309	87.2	5,610	74.3
Age at identification of IPV				
19–29	976	36.9	2,467	32.7
30–39	863	32.6	2,532	33.6
40–54	809	30.6	2,548	33.8
Education				
Primary	879	33.3	2,128	28.2
Upper secondary	1,402	53.1	4,209	57.0
Tertiary	362	13.7	1,104	14.8
Occupation				
Employed	1,256	47.5	4,189	55.5
Non-employed	1,387	52.5	3,352	44.5
Assault				
Physical assault	2,329	88.0	6,226	82.5
Personal offense ^a^	1,125	42.5	1,247	16.5
Menace	1,014	38.3	1,049	13.9
Sexual offense	87	3.3	74	1.0
Relationships				
Current marriage or co-habitant partner	1,516	57.3	4,393	58.2
Living conditions				
Cohabiting with suspect at the initial IPV	1,132	42.8	3,555	47.1
Mental disorder diagnosed before the initial IPV event (previous 12 months)				
Any mental disorders ^b^	934	35.3	2,034	27.0
Substance use disorders (SUD)	277	10.5	494	6.6
Depression	311	11.7	698	9.3
Anxiety	270	10.2	593	7.9
PTSD	33	1.3	74	1.0
Prevalence of individuals with at least one mental healthcare contact in secondary and primary care based on ICD-10 diagnoses (previous 12 months) ^c^				
Secondary care	508	19.1	1,041	13.8
Primary care	477	18.0	1,082	14.3

a Personal offense includes robberies, deprivations of personal liberty, menaces, and persecution.

b Any mental health disorders include secondary, primary care based on ICD-10 diagnoses and ICPC2.

c Individuals with mental healthcare contacts in both secondary and primary care were counted separately in each category.

Table 2 presents the pre- and post-event prevalence of any mental health service contact, stratified by group (multiple vs. single report) and gender. Among individuals with multiple reports, women showed a higher prevalence of mental disorders both before and after the event compared to men. The increase of prevalence was somewhat greater in the multiple report group than in the single report group across both genders.

**Table 2.** Prevalence (Pre- and Post-Event) of Individuals With at Least One Mental Healthcare Contact,^a^ Stratified by Gender (*N* = 10,195).

Group	Pre (*N*)	Pre (%)	Post (*N*)	Post (%)
Multiple reports (treatment)				
Men	103	30.4	122	36.0
Women	831	36.0	985	42.6
Single report (control)				
Men	477	24.6	553	28.5
Women	1,557	27.8	1,898	33.8

a Mental health contact includes secondary, primary care based on ICD-10 diagnoses and ICPC2. Each individual was counted only once in both the pre- and post- periods.

Table 3 presents the results of the DID with a linear probability model estimating the effect of revictimization on any mental health contact. At baseline, individuals with revictimization (the treatment group) had an 11.1% probability of utilizing mental health services. The estimates show a statistically significant increase in post- mental health service use of 0.9 percentage points, corresponding to a relative increase of 8.0% (β = .009, 95% CI [0.001, 0.017], *p* = .029). When we conducted gender-stratified analyses, significant results were observed only among women. After adjusting for covariates, no substantial changes were found; the results remained statistically significant, with a 0.9 percentage point increase in probability (see Supplemental Table 2).

**Table 3.** Crude DID Estimates of the Effects of Revictimization on Any Mental Health Contact, Based on Linear Probability Models, for Men, Women, and the Total Population.

Estimate	Total	Men	Women
Coefficient (DID) [CI]	0.009 [0.001–0.017]	0.000 [−0.019–0.019]	0.010 [0.001–0.018]
*p*-value	.029	.999	.036
Baseline	11.1%	9.1%	11.4%
% change	8.0%	12.4%	8.3%

*Note*. Coefficients from DID estimates (group × time) are reported. The baseline refers to the outcome variable measured for the treatment group over the previous 12 months. CI = 95% confidence interval; DID = difference-in-differences.

When adjusted for covariates in the gender-stratified model, the results remained the same. Both the crude and adjusted models showed statistical significance among women, whereas no significant association was found for men (see Supplemental Table 3).

**Figure 2.**
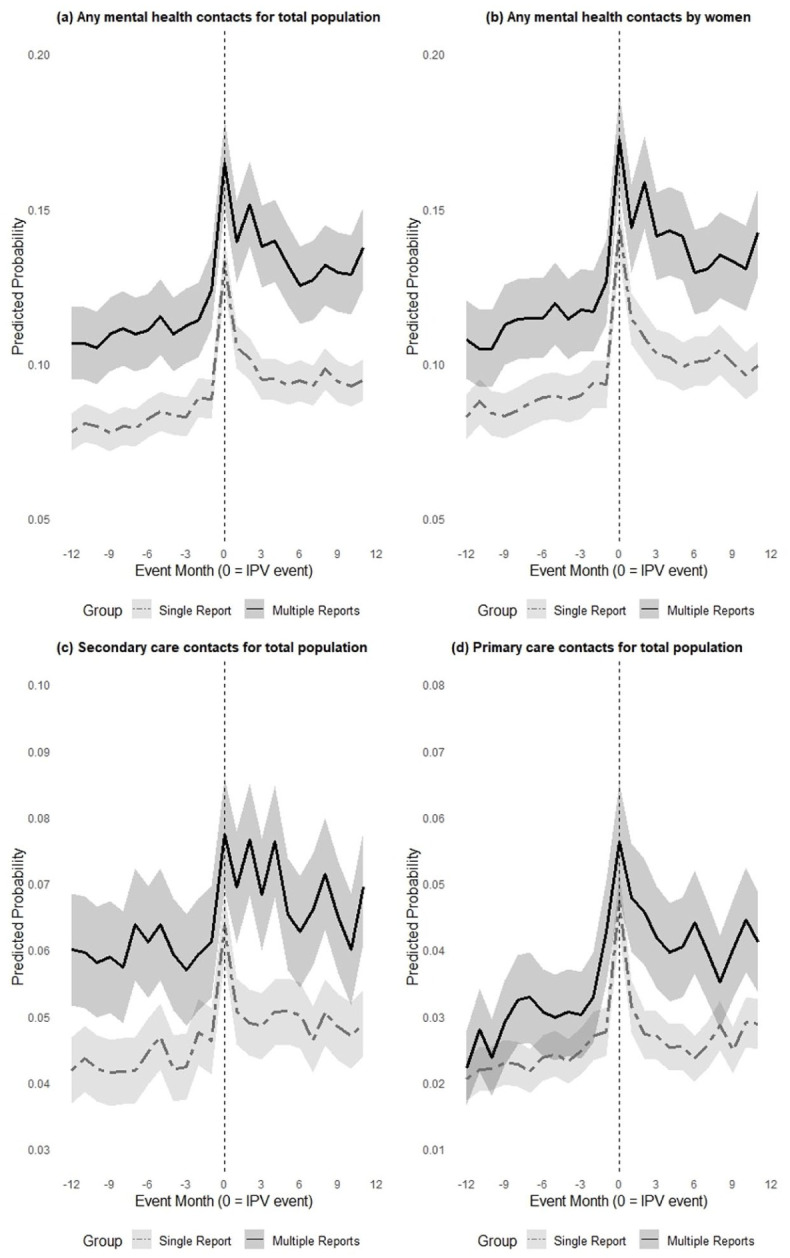
Predicted probability with 95% confidence intervals of service use by group: (a) any mental health contacts for total population, (b) any mental health contacts by women, (c) secondary care contacts for total population, and (d) primary care contacts for total population (based on ICD-10). Event month 0 includes the violent event.

Figure 2 shows the predicted probability of mental health service use by group, with 95% confidence intervals. Individuals with multiple reports of IPV consistently exhibited higher levels of mental health service utilization throughout the study period. Visual inspection suggests that the parallel trends assumption for any mental health contact was met during the pre-IPV event period (Figure 1(a)). As the majority of the sample consisted of women, the service use patterns for the total population closely mirrored those observed among women (Figure 1(b)).

The patterns for both primary care and secondary care services show greater variation in monthly probabilities over time; however, similar patterns are observed, with a sharp peak around the time of the IPV event (Figure 1(c) and (d)). Both primary and secondary care services consistently exhibited higher utilization among individuals with multiple IPV reports.

Table 4 presents the results of multivariable logistic regression models, adjusted for sociodemographic factors and stratified by gender, to investigate risk factors for IPV revictimization. Pre-existing depression (Model 1) was not a significant risk factor for IPV revictimization in either men or women. In contrast, SUD (Model 2) demonstrated considerably stronger and more consistent associations for both men and women. Among women, higher education levels and living with a suspect were associated with lower odds of revictimization, while non-employment was associated with higher odds; all three factors were statistically significant. However, among men, none of these factors were significantly associated with revictimization.

**Table 4.** Multivariable Logistic Regression Models of Associations Between Selected Mental Disorders and IPV Revictimization, Stratified by Gender.

Outcome	Predictors	Male victims		Female victims	
		*OR*	CI	OR	CI
(Model 1)					
Revictimization	Depression	1.16	[0.73–1.84]	1.14	[0.98–1.33]
	Age (ref: 19–29)				
	30–39	1.06	[0.79–1.43]	0.92	[0.81–1.03]
	40–54	1.00	[0.74–1.34]	0.90	[0.80–1.02]
	Education (ref: primary)				
	Upper secondary	0.87	[0.68–1.13]	0.83**	[0.73–0.93]
	Tertiary	0.84	[0.53–1.33]	0.82*	[0.69–0.97]
	Occupation (ref: employed)				
	No employment	1.09	[0.85–1.39]	1.29**	[1.16–1.43]
	Living with a suspect				
	Yes	1.01	[0.80–1.28]	0.85**	[0.77–0.94]
(Model 2)					
Revictimization	Substance use disorders (ref: without substance use)	1.99***	[1.43–2.78]	1.64***	[1.36–1.98]
	Age (ref: 19–29)				
	30–39	1.02	[0.76–1.38]	0.90	[0.80–1.02]
	40–54	0.97	[0.72–1.30]	0.88*	[0.78–1.00]
	Education (ref: primary)				
	Upper secondary	0.90	[0.69–1.16]	0.84**	[0.74–0.94]
	Tertiary	0.89	[0.56–1.40]	0.84*	[0.71–0.99]
	Occupation (ref: employed)				
	No employment	0.97	[0.75–1.25]	1.24***	[1.12–1.39]
	Living with a suspect				
	Yes	1.01	[0.80–1.28]	0.84**	[0.76–0.93]

*Note*. *OR* = odds ratio; CI = 95% confidence interval.

Significance levels: *p < .05. **p < .01. ***p < .001.

## Discussion

### Main Findings

Based on our register-based study, we found that IPV victims with multiple reports exhibited higher mental health service utilization than single report cases throughout the 2-year study period. The magnitude of the increase in mental health service utilization for IPV revictimization was approximately 8.0%, with an increase of 0.9 percentage points following the IPV event. Mental health service utilization peaked sharply around the occurrence of the IPV event for both groups. Pre-existing SUDs were significant predictors of revictimization for both men and women.

Our study offers new insights into the associations between IPV revictimization and mental health service utilization before and after a recent police-reported IPV incident. The sharp peaks in mental health service use around IPV incidents among individuals experiencing revictimization, among both genders, had not been well established in previous research. Our findings align with existing literature indicating a link between victimization/revictimization and increased mental health service use (Rasmussen et al., 2021; Sariaslan et al., 2024) and between pre-mental health conditions and victimization/revictimization among individuals with psychiatric symptoms (Bhavsar et al., 2019; Christ et al., 2022). Additionally, our results support previous evidence that SUDs are consistently identified as a significant risk factor for revictimization (Cole et al., 2008).

### Implications

These findings have important implications for clinical practice in identifying and responding to revictimization. When IPV is suspected, health professionals are obligated to strike a balance between maintaining confidentiality and ensuring patient safety and are legally responsible for documenting cases and providing holistic support. Meanwhile, they often face barriers such as limited knowledge, lack of training, or and poor organizational support (Hudspeth et al., 2022).

The rationales for increased mental health service use among revictimized individuals are not entirely clear; one possible explanation is that ongoing traumatic experiences may worsen mental health (Charak et al., 2020). IPV victims often expect health professionals to do more than simply listen, such as offering referrals (Tarzia et al., 2020).The relatively high prevalence of SUDs may reflect coping strategies (Afifi et al., 2012), even though SUDs are associated with an increased risk revictimization and poorer treatment outcomes (Ogden et al., 2022). While intervention strategies were beyond the scope of this study, our findings highlight the importance of clinical awareness of revictimization among individuals with complex mental and social needs. For example, individuals with SUDs may resist support due to these underlying factors. Recognizing these complex needs may help clinicians conduct more effective IPV screening, provide better trauma-informed care, systematically make treatment referrals, and support long-term engagement (Ogden et al., 2022).

The prevalence of mental disorders among both groups in our study is higher than that of the general population in Finland (16%; OECD/European Observatory on Health Systems and Policies, 2023). Interestingly, our results showed that individuals with revictimization already exhibited higher mental health service utilization during the pre-IPV period. This suggests that they may have been exposed to IPV or traumatic experiences before the study period. Furthermore, while the DID results were statistically significant, they did not indicate a dynamic change in mental health service use over the post-IPV period, as observed from the trend in the figures.

Considering alternative methods, it might be possible to compare individuals who experience violence in a later year. However, this approach is problematic, as individuals with similar sociodemographic or clinical characteristics may differ in unobservable factors, such as resilience skills. Additionally, their likelihood of accessing police or other formal services may vary if they are exposed to violence in close relationships. Furthermore, differences in service use patterns observed in our study may also reflect variations in data sources. Studies conducted in both in Finland and internationally (Kennedy et al., 2023; Papalia et al., 2024; Siltala et al., 2023) have shown that IPV populations identified through police data had some overlaps and different characteristics from those identified through health records. These complexities underscore the challenges in accurately estimating the effects of IPV on health service utilization.

This study identified a temporal association between mental health service use and revictimization. While we applied a DID framework, causality remains unclear due to potential unobserved confounders, such as earlier violence experience. Although pre-existing mental health conditions may increase vulnerability to revictimization, only SUDs showed a statistically significant association with revictimization. Thus, while a bidirectional association between violence and mental health has been reported in the literature, its plausibility remains uncertain in our results. Furthermore, our study population, characterized by lower educational attainment and non-employment, highlights the roles of social causation (e.g. poor socioeconomic conditions contributing to adverse mental health outcomes) and health selection (e.g. poor mental health influencing socioeconomic outcomes) (Hakulinen et al., 2023). The overrepresentation of individuals facing socioeconomic disparities underscores the association between these factors and IPV exposure, which may exacerbate existing mental health conditions.

### Strengths and Limitations

A key strength of our study is the use of a unique, linked Finnish nationwide administrative dataset. Finnish health registers are considered to be of high quality for research (Laugesen et al., 2021; Sund, 2012; Suokas et al., 2024), and the linked dataset provides comprehensive details on the study population, including background information, the exact date of the reported IPV event, and primary and secondary healthcare information. Although we cannot determine the exact occurrence of the violent incidents, our methodology enables us to distinguish between single and multiple reports and establish a baseline of at least 1 year free from violence reports in police records. Furthermore, register-based data facilitate the tracking of mental health trajectories over time, which is difficult to capture through survey-based studies alone.

Despite its strengths, our study has several limitations that should be acknowledged. First, the study population was derived exclusively from police reports, suggesting that our sample may consist primarily of more serious cases of physical IPV compared to unreported incidents. The extent of collaboration between police and mental health services is context-dependent and may vary across regions or countries. Therefore, our findings may not be generalizable to all individuals exposed to IPV. Second, although Finnish healthcare records provide accurate data on visits, admissions, and discharges, our study period is relatively short. As a result, we can only examine temporal associations between mental health service use, pre-mental health conditions, and revictimization. Additionally, individuals with a single report may have had pre-existing mental health conditions or prior IPV exposure before the study period. Third, private mental health outpatient care, particularly through occupational health services, constitutes a relatively substantial component of the Finnish healthcare system. However, our data did not include privately purchased outpatient care. Fourth, our study relied on diagnosis-based mental health records, which may capture a different aspect of mental health compared to self-reported mental health symptoms. While this enhances reliability, it may introduce selection bias, as IPV victims who report violence may systematically differ from those who do not. Not all individuals, particularly those experiencing revictimization, seek formal healthcare, as disclosing violence can be challenging (Gartland et al., 2022; Hullenaar & Frisco, 2020). Furthermore, we could not determine whether these IPV victims were referred to specialized services after the initial IPV event, introducing a potential confounding. Fifth, in this study, we consider gender as a binary variable based on the registered gender. We acknowledge that some individuals do not identify within the binary framework, and that others may identify differently from their registered gender. This limitation indeed points to the increased risk of violence among gender minorities, which remains an important area for future research. Lastly, our study did not include information on adverse childhood experiences, specifically childhood sexual abuse (Mazzarello et al., 2022; Papalia et al., 2021), which are significant risk factors for revictimization in adulthood. Future research will apply alternative methods to address adverse childhood experiences and extend the observation period to better assess the association of IPV with revictimization.

## Conclusion

To conclude, our study highlighted that IPV victims with revictimization use mental health services more frequently than those with single reports. This finding suggests that single incidents may reflect acute effects, whereas repeated violence victims may exhibit and amplify chronic or persistent mental health conditions. Understanding the characteristics of revictimization is essential for improving clinical responses to IPV. This requires not only sufficient professional training and knowledge but also systematic and organizational changes to strengthen competency in practice (World Health Organization, 2025). The pre-existing mental health and socioeconomic condition of individuals may serve as key opportunities for early identification and prevention.

There is a need to improve not only access to healthcare services for IPV victims but also ensure seamless coordination with a trauma-specific support system. When trauma-related experiences accumulate over time, disclosing IPV to formal services can be challenging, even when support is needed. Therefore, developing integrated services between healthcare, social services, and law enforcement is crucial to ensuring targeted and timely early intervention.

## Supplemental Material

sj-docx-1-isp-10.1177_00207640251379256 – Supplemental material for Revictimization and Mental Health Service Use in Intimate Partner Violence: A Comparison of Single and Multiple Reports Using Linked Police and Health Registers Supplemental material, sj-docx-1-isp-10.1177_00207640251379256 for Revictimization and Mental Health Service Use in Intimate Partner Violence: A Comparison of Single and Multiple Reports Using Linked Police and Health Registers by Tomomi Hisasue, Marie Kruse, Johanna Hietamäki, Jani Raitanen, Visa Martikainen, Kimmo Suokas, Pekka Rissanen and Sami Pirkola in International Journal of Social Psychiatry
