# Supplementary material for: Revictimization and Mental Health Service Use in Intimate Partner Violence: A Comparison of Single and Multiple Reports Using Linked Police and Health Registers
Source: Int J Soc Psychiatry. 2025 Oct 9;72(3):668–78. doi: 10.1177/00207640251379256 (PMC13121816; doi:10.1177/00207640251379256)
Supplement: sj-docx-1-isp-10.1177_00207640251379256 – Supplemental material for Revictimization and Mental Health Service Use in Intimate Partner Violence: A Comparison of Single and Multiple Reports Using Linked Police and Health Registers [file sj-docx-1-isp-10.1177_00207640251379256.docx]

Table S1. Diagnostic codes for assessing health service use

| **Variable** | **ICD-10 diagnostic code** |
| --- | --- |
| Any psychiatric disorder | F00–F99 |
| Substance misuse disorder | F10–F19 |
| Depression | F32–F39 |
| Anxiety | F40–F42, F44–F45, F48 |
| Post-traumatic stress disorder | F43.1 |

Equation 1: The DID model specification

Our model is a standard 2x2 DID approach:

$Y_{it}=\beta_{0}+\beta_{1}Repeat_{i}+\beta_{2}Post_{t}+\beta_{3}\left( Repeat_{i}*Post_{t} \right)+ \beta_{4}X_{i}+ \epsilon_{i}$ (1)

where $Y_{it}$ is the outcome of interest, mental health service use for IPV victim $i$ at time $t$. Time was measured as either pre- and post or monthly intervals. Repeat is a binary variable that indicates whether a victim is categorized into the multiple reports group (treatment group) or the single report group (control group). Post is a binary variable, where 0 indicates the period before the IPV event and 1 indicates the period after the event. The Repeat * Post interaction term captures the DID effect of revictimization on health services utilization after the IPV event. $X_{i}$ is a vector of covariates including age, gender, and sociodemographic factors. The parallel assumption of an event study of our model (1) is assessed by visual inspection and the lines appear to be roughly parallel for pre-IPV periods. The parameters were estimated using the linear probability model (ordinary least squares).

Table S2. DID estimates of effects on any mental health contacts with linear predicted model

|  | Total | |
| --- | --- | --- |
|  | Unadjusted | Adjusted |
| Any mental health service use | |  |
| Coefficient (DID)  [CI] | 0.009  [0.001–0.017] | 0.009  [0.001–0.017] |
| p-value | 0.029 | 0.029 |

Any mental health contact includes secondary, primary care based on ICD-10 diagnoses and ICPC2.

Coefficients of DID estimates (group*time) are reported.

Adjusted for age, gender, education, and employment

CI: 95% confidence interval, DID: Difference-in-differences

Table S3. DID estimates of effects on any mental health contacts with linear predicted model stratified by gender

|  | Men | | Women | |
| --- | --- | --- | --- | --- |
|  | Unadjusted | Adjusted | Unadjusted | Adjusted |
| Any mental health service use | |  |  |  |
| Coefficient (DID)  [CI] | 0.000  [-0.019–0.019] | 0.000  [-0.019–0.019] | 0.010  [0.001–0.018] | 0.009  [0.001–0.018] |
| p-value | 0.999 | 0.987 | 0.036 | 0.036 |
| Baseline | 9.1 | - | 11.4 | - |
| % Change | 12.4 | - | 8.3 | - |

Any mental health contact includes secondary, primary care based on ICD-10 diagnoses and ICPC2.

Coefficients of DID estimates (group*time) are reported. The baseline is the outcome variable measured for the treatment group over the previous 12 months.

Adjusted for age, education, employment cohabitation

CI: 95% confidence interval, DID: Difference-in-differences
